# Challenges in Medical Supplies Management in Community Healthcare Services during the Covid-19 Pandemic in Thailand

**DOI:** 10.30476/IJCBNM.2021.93298.1916

**Published:** 2022-04

**Authors:** Praditporn Pongtriang, Atsarayut Matkaew

**Affiliations:** 1 Department of Adult and Elderly Nursing, Faculty of Nursing, Suratthani Rajabhat University, Surat Thani, Thailand; 2 Department of Technology in Industrial Management and Logistics, Faculty of Industrial Technology, Nakhon Si Thammarat Rajabhat University, Nakhon Si Thammarat, Thailand

## DEAR EDITOR

With high infection rates in many countries and more than four million deaths globally, ^1^ the coronavirus disease (COVID-19) pandemic has adversely affected healthcare services and providers, exacerbating their workload and risk of infection. ^2^ Due to insufficient capacity, medicines, and medical supplies, ^3^ many countries have struggled to maintain healthcare activities and implement prevention strategies—a barrier to achieving the expected health outcomes for their population.

Thailand is one of the developing countries confronting a problematic situation since the first wave of the Covid-19 outbreak. With the implementation of public health restrictions curtailing everyday life and other laws and regulations to control the crisis—including measures such as state quarantine, social distancing restrictions, and area lockdowns—community health services have been struggling to manage the imbalance between the demand and supply of resources, especially medical supplies essential for infection prevention, and most importantly, the safety of healthcare providers working at the frontlines, ^4^ such as surgical masks, gloves, personal protective equipment, alcohol sanitization products, and viral screening toolkits. ^5^ The potential risk of infection needs to be minimized to avoid a shortage of healthcare professionals in the Thai community. Following the pandemic, many community hospitals in Thailand were forced to expand their capacity to fight the rise in infections. However, the business continuity planning systems in primary and secondary healthcare facilities in Thailand have proved insufficient to contribute to the supply chain and logistics management for compliance. 

In this context, this article formulates strategies that could alleviate the medical supply shortage in community health services in Thailand. First, the engagement of community health activities is essential to be determined for managing the demand and supply ongoing crisis. Due to the health policies changing regularly, community nurses require to develop the planning and execution of preventative activities across the community. Second, analyzing the context of the problem could form a basis for information capable of encouraging the flow of managing a shortage situation in a different context. Lastly, establishing a community data system for epidemiology and service resources is essential for expanding the community-based quarantine capacities. Informational technologies are also a part of the workforce during a critical situation. However, there are several limitations in practice in the community including a lack of information and communication technology (ICT) expertise and a barrier of connection. Such limitations require long-term planning coupled with a community context analysis.

Community nurses play an important role in medical supply management and the smooth running of the organization. They craft reports, engage in reflection, and take necessary action. In the context of the medical supply shortage, nurses need to make fast decisions to manage and adjust to the situation. In the face of the pandemic, the most effective strategy to manage the shortage of medical supplies and human resources could be multidisciplinary teamwork. Community volunteers can offer prevention assistance in communities and thus become the key to successful running of healthcare operations in several dimensions. However, with the current limitations, it is a challenge for community nurses, who are in charge of managing and supporting the healthcare system for Thai citizens.

This article highlights the challenges the healthcare system is struggling with due to the pandemic and offers possible strategies to deal with it. Though every country has its different context and situational severity, a critical solution and an in-depth understanding of the situation in real-time for eliminating the adverse effect of the pandemic are crucial to saving lives. 

## ACKNOWLEDGEMENT

The authors thanks for supporting by Suratthani Rajabaht University and Nakhon Si Thammarat Rajabhat University.

**Conflict of Interest:** None declared
